# Quantum Key Distribution for Critical Infrastructures: Towards Cyber-Physical Security for Hydropower and Dams

**DOI:** 10.3390/s23249818

**Published:** 2023-12-14

**Authors:** Adrien Green, Jeremy Lawrence, George Siopsis, Nicholas A. Peters, Ali Passian

**Affiliations:** 1 Department of Physics and Astronomy, The University of Tennessee, Knoxville, TN 37996, USA; agreen91@vols.utk.edu (A.G.); siopsis@tennessee.edu (G.S.); 2 Electric Power Research Institute, Charlotte, NC 28262, USA; jlawrence@epri.com; 3 Quantum Information Science Section, Computational Sciences and Engineering Division, Oak Ridge National Laboratory, Oak Ridge, TN 37831, USA; petersna@ornl.gov

**Keywords:** quantum key distribution, QKD, quantum security, hydropower, dams, QKD post-processing, critical infrastructure, cyber-physical security

## Abstract

Hydropower facilities are often remotely monitored or controlled from a centralized remote control room. Additionally, major component manufacturers monitor the performance of installed components, increasingly via public communication infrastructures. While these communications enable efficiencies and increased reliability, they also expand the cyber-attack surface. Communications may use the internet to remote control a facility’s control systems, or it may involve sending control commands over a network from a control room to a machine. The content could be encrypted and decrypted using a public key to protect the communicated information. These cryptographic encoding and decoding schemes become vulnerable as more advances are made in computer technologies, such as quantum computing. In contrast, quantum key distribution (QKD) and other quantum cryptographic protocols are not based upon a computational problem, and offer an alternative to symmetric cryptography in some scenarios. Although the underlying mechanism of quantum cryptogrpahic protocols such as QKD ensure that any attempt by an adversary to observe the quantum part of the protocol will result in a detectable signature as an increased error rate, potentially even preventing key generation, it serves as a warning for further investigation. In QKD, when the error rate is low enough and enough photons have been detected, a shared private key can be generated known only to the sender and receiver. We describe how this novel technology and its several modalities could benefit the critical infrastructures of dams or hydropower facilities. The presented discussions may be viewed as a precursor to a quantum cybersecurity roadmap for the identification of relevant threats and mitigation.

## 1. Introduction

**Figure 1:**
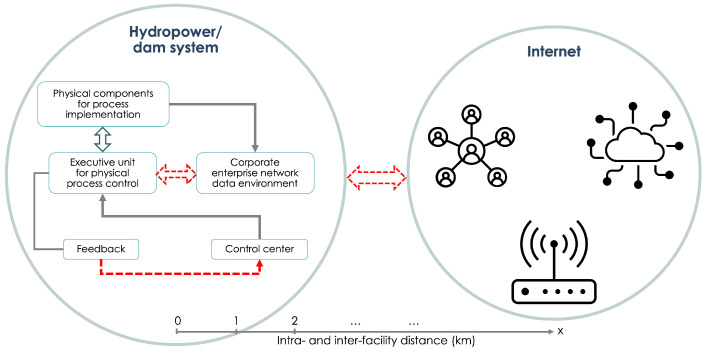
It is widely recognized that the existing hydro infrastructure has cybersecurity weaknesses that can be exploited at both intranet (**left**) and internet (**right**) levels. As exemplified by the dashed arrows, cyber problems are ultimately due to a lack of secure communications and the presence of side channels among the various components/devices inside and outside the system.

Security of critical infrastructures poses a complex and dynamic problem teeming with loopholes, weak links, and outdated measures that create an array of cyber vulnerabilities and safety concerns [1,2,3]. Innovative solutions are needed to protect existing and developing infrastructure (see Rass et al. [1] for what constitutes a “critical infrastructure” and related discussions). Currently, in the US alone, less than 3% of the 80,000 dams produce power. Efforts to generate more clean power from these existing dams mean the utilization of advanced technologies and modernization. Therefore, digital technologies are expected to continue to be integrated with hydroelectric projects (including fleet modernization). The gain (e.g., in the efficiency from turbines and generators) that comes with digitalization and the use of advanced information and communication technologies benefit the missions and objectives of an increasing number of stakeholders in hydro energy. These efforts mean increased connectivity (e.g., enhanced remote control and monitoring of the operational conditions of the assets). Higher connectivity is also expected from optimization efforts to operate neighboring hydropower facilities across whole river systems. Predictive and intelligent maintenance [4], higher efficiency operation, development of digital twins, etc., all require communication of measurement results and associated data analysis from many components and equipment, often in real time. Higher connectivity, that is, a larger number of communications channels, means a larger cyber-attack surface, and consequently, more risks, as depicted in Figure 1. A brief summary of some of the basic security issues, and a simplified encryption example are provided in Table 1, and Appendix A, respectively; see also relevant discussions by Ratnam et al. [5]. In what follows, for convenience, some relevant terms invoked are defined in Table A1.

Clearly, the noted risks associated with exploiting the weaknesses of communications channels need to be addressed. However, known classical (non-quantum) encryption techniques cannot eliminate such risks (for a simple classical encryption example, see Appendix A). This is because, to protect the confidentiality of the communicated messages, classical security utilizes the mathematical complexity of classical cryptography techniques and projections of computing technology development as opposed to quantum approaches where the security is not based on technology assumptions. Attempts to intercept or read off the quantum information will disturb the fragile quantum states carrying the information. Fundamentally, there is no amount of care one can exercise that would enable this process without creating a detectable quantum disturbance. This property can be used to distribute keys that are secure regardless of computing power which have the potential to enable the long-term security of hydropower and dam infrastructure.

Traditional encryption is currently used to validate the legitimacy and authenticity of the sender and receiver, while also obfuscating the information. This means that even if communication is intercepted [8], it cannot be read or understood unless the attacker has the decryption key. Traditional methods rely on secure creation and exchanges of keys to ensure end-to-end protection. Current potential problems with encryption include incorrect implementation of encryption in software (vulnerabilities), attacks against the users, supply chain attacks, compromise of the keys, brute forcing the message, or analyzing the encrypted communication to derive the key. With the future advancement of quantum computing, the speed at which brute force attacks can successfully decrypt communications (using many current algorithms) could render present encryption insufficient. Attempts to evade security measures including cyber, malware, and side-channel attacks may generate results [1]. Reported attacks on dams and other critical infrastructure have revealed significant cybersecurity gaps and problems in existing infrastructure, which is ultimately due to a lack of secure communications channels. Physically, the two primary channels over which information, commands, and instructions are conveyed/exchanged are either optical fiber or free space. Both of these channels can be exploited by attackers to threaten the assets. This article proposes a solution and how it may be applied to this problem. Clearly, any solution should be compatible with the rapidly growing communication technologies encompassing edge computing and sensing [10,11], IIoT (the Industrial Internet of Things), IoE (the Internet of Energy), etc. Such a solution must position the hydro security infrastructure for resiliency against increasingly advanced and sophisticated attacks.

Emerging quantum technologies promise to help solve the security problem of communications channels. The most well-established quantum communication technology is currently quantum key distribution (QKD), which has been shown to achieve information-theoretic security (ITS), meaning it does not rely on any technology assumptions, such as what problems are difficult to compute. Such a solution has already been demonstrated in the form of the deployment of state-of-the-art QKD-based communication technologies across the electric grid [12,13,14,15]. To date, as technology transitions from research labs to the commercial sector, only a few commercial QKD systems have made their way to the market. An evaluation and comparison of all QKD modalities against the hydropower system’s requirements is needed (see Table 2). However, in describing our QKD-for-hydro use case, we note that technologies such as Quantum Digital Signatures (QDS) [16] and Quantum Secret Sharing (QSS) [17] play important roles in ensuring communication integrity and the secure distribution of sensitive data among multiple stakeholders, respectively. These modalities, along with Quantum Secure Direct Communication (QSDC) [18,19], provide robust frameworks for safeguarding critical communications. Additionally, the concept of physical layer security, as explored in Rothe’s works [20], offers another layer of protection by leveraging the physical properties of the communication medium itself to enhance security.

Logically, one may categorize noise sources in QKD operating in a hydropower environment into two main categories: those more fundamental than what occurs in that specific environment, as listed in Table 3, and those specific to it, as listed in Table 4, with related discussions elsewhere [23,24,25,26,27].

While noise sources independent of the environment may be addressed with advances in technology and improved equipment, those induced by the dam environment may require specialized solutions tailored to the unique challenges posed by such a setting. Separating these categories could help in better understanding and mitigating the noise sources.

Prior to describing our main objective, we note that the application of QKD in hydropower and dam facilities is driven by their specific operational characteristics and security challenges, notably the need for secure remote operations and the integration of cyber-physical systems. This focus differentiates their security requirements from those of other critical infrastructures such as nuclear sites, where physical security plays a more dominant role [33]. Also noteworthy in this focus is that the convergence of QKD with advanced sensing technologies presents a comprehensive approach to cybersecurity and physical integrity. Examples include the emergence of metadevices, as discussed by Ijaz et al. [34], or the development of chip-based QKD systems [35], highlighting the potential of metamaterials and metasurfaces in enhancing data transmission and imaging systems, which could be pivotal in monitoring and communication processes within hydropower facilities. Additionally, the advancements in high-sensitivity force sensors based on novel materials, as reviewed by Zhang et al. [36], underline the importance of precise environmental and structural monitoring, an essential aspect for the physical security of dams. Furthermore, the application of quantum squeezing techniques, as elucidated by Wang and Zhang [37], indirectly enriches the QKD quantum technology toolsets employed for ensuring robust cybersecurity.

## 2. Objective

**Figure 2:**
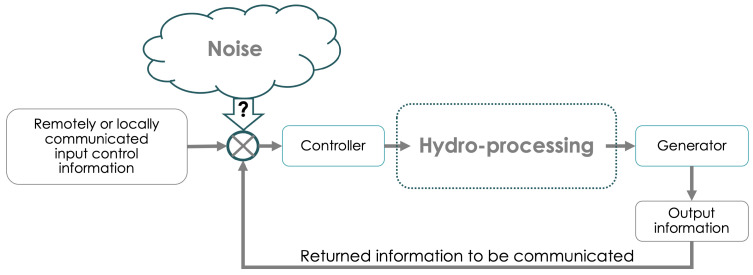
Schematic depiction of feedback loop for control of the hydro-electric process. Multiple points of vulnerabilities can be readily identified.

**Figure 3:**
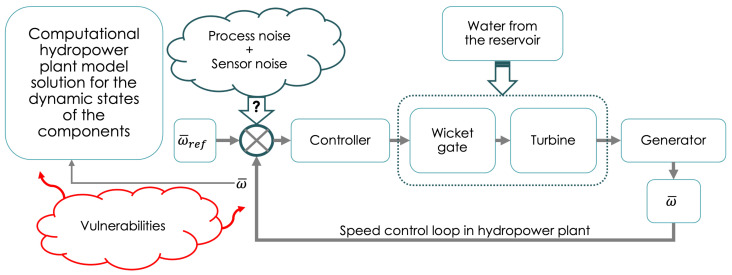
An example of side-channel threats in a hydropower plant. Secure speed control of the generator is critical for safe operation. During remote control, cyber-attacks may alter the commands, leading to altered input values for the speed control of the generator. A set of differential equations (see Chandra et al. [39]), describing the time dependence of the states, including frequency change $\Delta \bar{\omega }$, turbine water velocity ${\bar{u}}_{t}$, gate opening $\bar{g}$, and the pilot actuator position variation $\Delta {\bar{x}}_{e}$, are solved in a plant model. The solutions emphasize the many parameters and functional dependencies of the hydropower plant model. The integrity of the signal returned by the feedback loop is important to minimize vulnerabilities. These communications channels can be made secure using a future implementation of internet-level, intranet-level, and even “device-level” QKD.

Our objective is to elucidate the utility of QKD for protecting hydropower assets and articulate what quantum security technologies can bring to improve critical hydro infrastructure security. As simplistically depicted in Figure 2, a hydropower system is composed of many networked sensors, control systems, and operators that need to communicate with each other over geographically diverse locations. A relevant use case of secure communications may therefore be the implementation of a communications channel between the control room and an equipment controller. This would not only prevent cyber-attacks but also any side-channel attacks. The communication link corresponding to this connectivity can be made secure with long-term security provided by QKD. Any information to be exchanged between a party on the internet and a party on the dam network requires encryption or authentication. QKD can share unique private keys that can be used for this purpose (for a simple quantum encryption example, see Appendix A). Therefore, the generation of secure keys over the communication link is the first step (see Figure 3). Reaching a working understanding of the technical layout specific to a hydro facility could begin by building on previous work in carrying out cyber technical risk assessments, including that of hydropower systems and dams [13,38], and the development of a holistic cybersecurity risk reduction framework for fossil generation facilities [38], as well as the deployment of quantum communication for grid security, such as in [13,14,15]. Given that there are many such plant- and systems-level examples of a cyber threat to physical devices of the power plant, identifying a reasonable location in a dam or an equivalent testbed to implement the QKD is prudent. This important step lays the foundation for developing a similar use case methodology that can be applied across various hydro facilities. One may envision research and development of a security use case taxonomy that can serve the broader energy infrastructure landscape. Such a taxonomy should be of direct benefit to the stakeholders since planning, marketing, energy distribution, customer privacy, service quality, and many other aspects of energy economics can be impacted by a better understanding of the security risks involved.

QKD is currently at the forefront of innovative communication technologies and is typically advertised as the next-generation security technology that, unlike conventional techniques, does not expire when bigger computers are built. QKD has been demonstrated for securing communications between parties on Earth [40] as well as between Earth and satellites [41,42]. To implement QKD, we first note that there are different modalities and protocols for QKD. Despite still being subject to intense research, new results are leaving research labs, and commercial systems are entering the market. For a brief description of the QKD protocols and modalities versus the specific requirements of the present use case, see Table 2 (see also the recent survey by Sharma et al. [43]).

## 3. Quantum Key Distribution and Its Implementation

In 1984, the BB84 protocol was introduced by Bennett and Brassard [23]. Today, BB84 is just one instance of many possible QKD protocols that harness quantum mechanics to share private keys. As an example of a standard implementation, the polarization of individual photons can be used to encode information. In BB84, the simplest, oldest, and most developed protocol, Alice sends single photons encoded randomly from a predetermined set of polarization states to Bob. As quantum states are disturbed when measured, any measured noise is attributed to an eavesdropper, resulting in an increase in the quantum bit error ratio (QBER), defined as:

(1) $$\mathrm{QBER}=\frac{\mathrm{number}\;\mathrm{of}\;\mathrm{error}\;\mathrm{bits}}{\mathrm{total}\;\mathrm{number}\;\mathrm{of}\;\mathrm{bits}\;\mathrm{exchanged}}.$$

If the QBER is low enough, the two parties can distill a shared, private key. For many QKD protocols, the process to distill a secure key involves four main steps: raw key exchange, sifting, error correction, and privacy amplification, as shown in Figure 3. Specifically, in the BB84 protocol, after the raw key is shared through the quantum channel, the sifted key is generated by Bob announcing, over a public classical channel, the basis he used to measure each photon. Alice then compares this with her basis choices. The bits corresponding to mismatched bases are discarded, resulting in the sifted key. Any QKD protocol will have errors in the sifted key due to the experimental imperfections and potential eavesdropping, which are corrected using an algorithm over the authenticated public channel. Finally, privacy amplification is also performed over the public channel to minimize any potential public information about the key. Generally, QKD can be divided into discrete variable (DV) and continuous variable (CV) implementations. DV encoding, such as the polarization example above, involves detecting single quantum states with direct detection single-photon detectors, and results in discrete measurement data. In continuous variable encoding, homodyne detection is used to measure continuous variables of quantum light, namely phase and amplitude, which carry information between Alice and Bob. CV-QKD protocols generally have the potential for higher key generation rates than DV protocols, especially in the presence of low-loss channels, as homodyne detectors do not have the significant dead time of direct detectors. However, CV protocols, especially in high-loss scenarios, face challenges related to error reconciliation, given the Gaussian noise characteristics of their keys [23]. A benefit of CV protocols is that they can be implemented in shared fiber with classical communication systems without the destruction of the quantum signal [44]. QKD can also be divided into entanglement-based and prepare-and-measure implementations. In many entanglement-based QKD protocols, entangled states of light are generated and shared between Alice and Bob. This can be performed by a third party or by one of the participants, such as Alice, who then sends one of the entangled particles to Bob. On the other hand, prepare-and-measure protocols, like BB84, involve Alice preparing a quantum state and sending it directly to Bob. For distances relevant to hydropower dams, prepare-and-measure protocols are currently the most practical and achieve the highest speeds [45]. In practical QKD implementations, information is often encoded into weak coherent pulses, such as those from faint lasers. While single-photon or entangled photon sources are ideal for QKD, their practical implementation can be challenging. The decoy state protocol allows weak coherent sources to be used effectively by mitigating the photon number splitting attack, making it possible to utilize attenuated lasers for quantum light sources without compromising security [46]. This encoding method has evolved to what is called decoy state QKD because certain laser pulses will act as decoys to test for the photon number splitting attack. Given that decoy state BB84 is among the most developed protocols, it is currently the most widely used QKD protocol.

**Figure 4:**
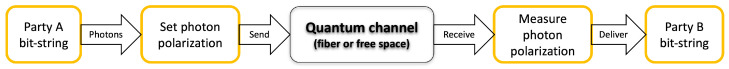
Schematic depiction of QKD. Two parties generate random bit-strings that are encoded in light pulses. The pulses are sent off via fiber or free space to a receiving party then are measured and converted to bit-strings. A message may be encoded via variations in the polarization of the light, as shown in the second box from left, or in its phase (see R. Wolf [49] for a formal introduction to these operations).

Studies of scientific and technical issues surrounding the security of the practical implementation of QKD have illuminated the possibility of various conceivable side-channel attacks. Realistic expectations from the performance of QKD subsystems mean that QKD must be carefully implemented [47,48] to avoid, for example, Trojan horse and photon splitting attacks. These attack scenarios are difficult to mount on practical QKD systems as they mature. The implementation of QKD begins by building its physical arrangement, which is composed of light sources, optical components to manipulate light, detectors, data acquisition, and processing electronics. The information encoded by doing a bit-by-bit exclusive “OR” with these keys will be secure if the key is kept private, only used once, and the key is larger than the message size. Successful implementation of QKD is measured by generating keys using the physical realization of the diagram shown in Figure 4.

**Figure 5:**
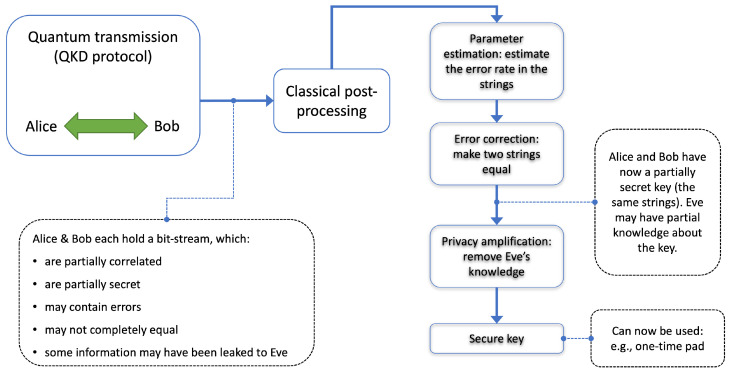
In the basic form of QKD, the two communicating parties, Alice and Bob, carry out quantum optical operations to distribute correlated bit-strings. After quantum operations are completed, post-processing is required. These include applications of various mathematical and numerical processing to generate the final keys. If an eavesdropper (Eve) attempts to intercept the quantum stage of the process, such attempts increase errors in the data. See Table 5 for further description and [49] for details.

The key questions to be answered are where QKD can be deployed in a hydropower communications network, and how it can be integrated with existing command and control interfaces. Of importance is the frequency of communications and the requirements of QKD-based one-time-pad (OTP) approaches to meet this need, in addition to communication latency requirements. A summary that systematically and collectively presents an overview of QKD (as depicted in general in Figure 4 and Figure 5) as well as it’s application to the hydroelectric domain will help to lead the wider utilization of quantum security in hydro infrastructures. Our goal is stimulate the production of this document, which would also complement those focused on classical cybersecurity.

## 4. Approach

An approach to the security of hydropower assets based on quantum technologies should naturally be relevant to other energy infrastructure security science and technology. Therefore, a high degree of connectivity is expected amongst several works in the energy research portfolio. For example, in fossil power plant cyber security, some previous investigations focused on (1) assessing how cyber risk changes across a facility’s life cycles, (2) performing consequence analysis to prioritize high-consequence events, (3) identifying the digital asset attack surface in sensors, instrumentation, and control equipment, and (4) mitigating cybersecurity control or countermeasures [38]. Such reports [38], describe the current industry cybersecurity best practices in fossil generation that are based on the first principles for cybersecurity engineering. Another specific example is related to grid security, where previous work focused on:

- conducting an analysis of commercial QKD capabilities [12,50];
- conducting an analysis of smart grid security needs [51];
- identifying the highest-value security needs that can be met by QKD [52].

The performed analysis was based on the NIST Framework and Roadmap for Smart Grid Interoperability Standards [15], as described in the related report [53]. To achieve the technical objectives above, we may consider the following discussions. It is important to note that operational technology (OT) architectures used in hydropower control and safety systems present unique challenges and considerations from standard information technology (IT) deployment of QKD. OT systems rely on legacy equipment, proprietary and unique operating systems, specialized protocols, and unique architecture requirements. In addition to the end-use case identification for QKD in hydropower, it is important to consider the impact of these architectures and infrastructures on QKD.

### 4.1. Use Case for Quantum Security in Hydro

In creating a use case, previous experience, e.g., in performing a cybersecurity risk assessment of other architectures, may be leveraged to show the holistic security benefit of the QKD solution. Use cases include remote monitoring and control, remote sensor, and IIOT deployment. Critical communications which rely on strong authentication in hydropower include:

- securing remote interactive access (control, maintenance, and repairs);
- remote monitoring (remote sensors for control/safety/monitoring, remote monitoring only centers with unidirectional traffic);
- vendor monitoring;
- supply chain security (validation of the authenticity of software and supply chain communications).

Control systems and operational technology (OT) rely on specific protocols for communications between field devices, programmable logic controllers, management servers and workstations, and other control system components. Many hydropower facilities that were designed with SCADAs (supervisory control and data acquisition systems) [6,54] are being upgraded to distributed control systems (DCS) as hydropower facilities are undergoing component and digital modernization. The control systems must be carefully architected to provide reliability and safety. Latency and reliability of the communications are crucial in these applications and should be considered. A priority use case should:

- document how it capitalizes on the specific environment of the dam/hydro facility or hydro testbed from a security point of view;
- identify security benefits/disadvantages of the QKD relative to traditional methods, in the identified use cases;
- document how it highlights the practical (logistical) suitability/applicability of the QKD for implementation within the dam/hydro environment/testbed;
- document a reference architecture for deployment in the selected use case;
- identify operational impacts on QKD deployment;
- highlight how the use case contributes to the missions of hydropower research facilities.

### 4.2. Integration of the QKD System with the Hydro Communications System

QKD is a novel quantum-based cybersecurity tool that allows for the generation and secure distribution of truly random number streams. Field demonstration of QKD has been reported in the case of a real-world electric utility optical fiber network [13]. A “key” is simply a string of bits, that is, a sequence of 0 s and 1 s, and a “message” is in the form of a bit-string. The end goal here is the successful use of keys generated using QKD by the communicating parties. For example, when the two communicating parties share a private key, they can use that key to encrypt any messages they intend to send and decrypt any messages they receive. This encryption prevents eavesdropping from accessing any information in the messages. This could, for example, take the form of the complete set of communications needed for remote control of a dam, or communication for a SCADA system [54]. This would ultimately entail generating random bits that are supplied to a computer hard drive or memory, at two (or more) locations. These bits are then to be used for the encryption of the messages between the two locations. Optical fiber-based QKD is highly versatile as fibers are immune to electromagnetic interference, and mechanically flexible so that they can penetrate confined areas, elaborate machines, and devices. The QKD process begins with a quantum transmitter (typically referred to as Alice, as indicated in Figure 5). The sender will have to generate light and prepare it in a specific quantum state. These light pulses, representing bit-strings, are then sent into an optical fiber to travel to another location, where they can be detected by a quantum receiver (typically referred to as Bob, as indicated in Figure 5), at the other end of the fiber. After concluding the quantum operations between the two communicating parties, to generate the final keys, the bit-streams must be post-processed. After processing the keys, as shown in Figure 5, they can be used to protect the information between communicating entities (users, control systems, sensors, actuators, SCADAs, etc.).

**Figure 6:**
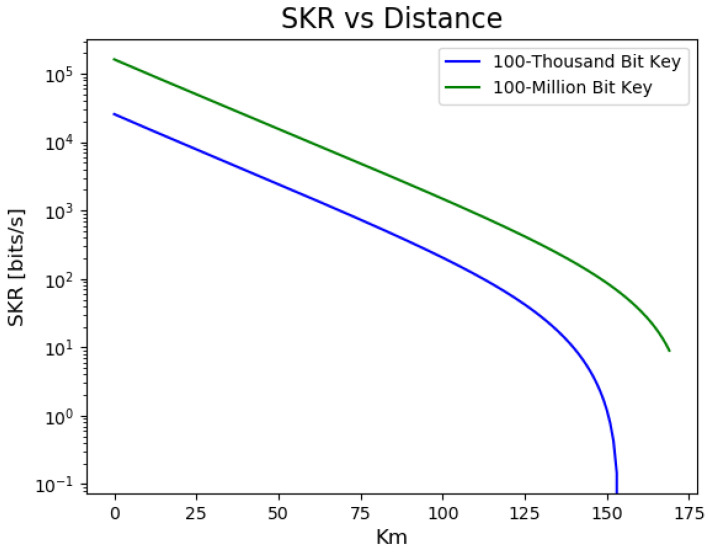
The final secure key rate as a function of the distance between communicating parties, derived from fiber-based loss and error models according to Equations (2) and (3). This representation assumes a decoy state BB84 variant of QKD, with parameters grounded in real-world and feasible experimental setups. As can be seen, for longer key lengths, the allowable communication distance before the SKR becomes impractical is reduced. An example of such a system, simulated at the University of Tennessee for a free-space deployment, is presented in [57]. Conventional devices, including a 1550 nm continuous wave laser and single-photon avalanche detectors, were employed in their work. At the rates shown, 100-thousand-bit key takes 4 s to distill and the 100-million-bit key takes 17 min.

The distributed keys are stored on a local computer where the encryption and authentication [12] may be implemented. The most computationally efficient (and therefore lowest-latency) encryption method remains the one-time-pad (OTP) method, where a message and key are combined with the exclusive OR operation (XOR). OTP exhibits ITS (information-theoretical security), i.e., it is secure regardless of an adversary’s computational power, with the following requirements: (1) the keys must be truly random, be kept secret, be are used once only, and (2) the message length is less than or equal to the length of the key. The resulting communications are then sent out through a classical transceiver. An experimental demonstration of relaying keys between relevant hydro infrastructure locations could conclude after a QKD operation over a given period (e.g., ∼hours). Such an experiment could implement QKD over a metro area distance (typical of hydro facilities) using a commercial QKD system. The main metric governing QKD system performance is the secret key rate (SKR), i.e., the (average) number of secret bits generated and distributed securely between parties per second. SKR, while largely dependent on the type of system and QKD protocol employed, is ultimately determined by the optical loss on a given fiber link. This loss γ, expressed in units of dB, is largely due to the fiber’s attenuation *a* dB/km, which typically arises due to absorption and scattering mechanisms and can be written for a fiber of length *L* km as γ=aL. It is crucial to minimize the losses, which also can be exacerbated by fiber-to-fiber connectors, sharp fiber bending, and splicing. High losses will reduce the throughput of the QKD process. The greater the optical loss, the lower the SKR, and vice versa. In situations where the optical link loss is significant, the SKR can be zero, indicating that no secret keys can be generated. From a practical standpoint, as noted above, optical losses receive contributions from two main factors: the physical distance along the fiber between two points (length attenuation) and splice, or connection losses. The former is indicative of intrinsic material losses in the optical fiber itself. Modern deployed optical fibers exhibit ≈ (0.2–0.5) dB/km for standard single mode fiber for telecommunications wavelengths around 1.3 to 1.6 μm. Modern fibers could have slightly lower loss than 0.2 dB/km, around 1.54 μm, but the inclusion of fiber-to-fiber connections, including in-field splices during deployment and patch cable connections within a communications facility or substation, can increase the average propagation loss. For this attenuation range, if, as simulated in Figure 6, the fiber is 175 km long, the total loss in the fiber will be in the range of γ=aL= (35–87.5) dB. Consequently, a viable QKD deployment must evaluate the optical link conditions between locations to see if it will allow for sufficient SKR for the desired operational requirements.

In QKD, the eventual length of the secure key is determined by several factors, including channel noise, error rates, and the specifics of the chosen protocol. While longer data collection times can yield larger secret keys, this could introduce delays before the key becomes available for encryption purposes. This is due to the need for post-processing steps like error correction, privacy amplification, and particularly the estimation of parameters such as the quantum bit error ratio (QBER) using a substantial portion of the sifted key. For real-world applications in hydropower plants, system optimization becomes vital. For instance, when several single-photon detectors in a command center are shared between remote links, the time each remote device utilizes a given detector should be optimized to reduce the total number of necessary detectors. This not only aids in efficient key generation but also in minimizing costs associated with hardware. The key rate or efficiency is not determined by a pre-selected length but rather emerges from the conditions of the quantum channel and post-processing. Practical QKD systems also need to address finite-size effects, where the security of the generated key can be influenced by statistical fluctuations. These effects become crucial in real-world applications such as hydropower plants, where reliable and timely key generation might be essential. An understanding of the communication frequency and topology between devices in such environments will be pivotal in tailoring QKD systems for optimal performance and cost-efficiency.

Figure 6 depicts how the SKR varies with distance for different key lengths, highlighting the impact of channel loss on the key rate. Similarly, Figure 6 illustrates the SKR’s sensitivity to misalignment angles in the system. The secure key rate (SKR), as derived from the theoretical framework introduced by Lim et al. [55], illustrates this dependency. In the protocol proposed by Lim et al., Alice sends Bob randomly polarized coherent states in two orthogonal bases: X and Z. While the X basis contributes to the secure key, the Z basis states are publicly disclosed to estimate the error rate in the X basis. The effective secure key length $L_{\mathrm{key}}$ is then described by:

(2) $$L_{\mathrm{key}}=s_{x,0}+s_{x,1}-s_{x,1}h\left(\phi_{x}\right)-{\mathrm{Leak}}_{EC}-6\log_{2}\left(\frac{21}{\epsilon_{\sec}}\right)-\log_{2}\left(\frac{2}{\epsilon_{\mathrm{cor}}}\right),$$

where $s_{x,0}$ and $s_{x,1}$ represent the number of dark counts and single-photon counts at Bob’s detector, respectively. The term $\phi_{x}$ denotes the error rate in the x basis. The binary entropy function $h(\phi_{x})$ [56] is given by:

(3) $$h\left(\phi_{x}\right)=-\phi_{x}\log_{2}\phi_{x}-\left(1-\phi_{x}\right)\log_{2}\left(1-\phi_{x}\right),$$

which captures the maximum information Eve can deduce about the total key given the shared bits used to determine the error rate. As such, the term $s_{x,1}h\left(\phi_{x}\right)$ must be subtracted from the total to yield a portion of the key that remains concealed from Eve. ${\mathrm{Leak}}_{EC}$ encapsulates the information exposed during error correction, while the concluding terms address finite-size effects. A deeper analysis, especially of terms rooted in the X basis signals and shaped by the sacrificed Z basis signals, is detailed in [55].

Figure 6 illustrates how the choice of key length, influenced by finite-size statistics, affects the secure key rate and associated generation time. Specifically, at a distance of 1 km, starting with the aim of distilling a 100-million-bit secure key yields a final key rate of approximately 98 kbps and takes 17 min to distill. In contrast, aiming for a 100-thousand-bit secure key results in a lower final key rate of about 25 kbps but only takes 4 s total to distill. Note that finite-size effects cause shorter key lengths to have higher uncertainty in the error and thus more bits are thrown away in post-processing, reducing key generation rates. In a continually operating secure communication system, these trade-offs highlight the importance of preemptive considerations. Factors such as communication frequency and average message size play a pivotal role in optimizing system performance and cost. Such metrics also influence choices regarding the QKD protocol, quantum encoding strategy, and equipment selection, ensuring that the system meets or exceeds the desired performance benchmarks.

The unique environment of a hydropower plant (see Table 4) introduces specific imperfections crucial in the context of a QKD system. Predominantly, additional loss and noise from such a setting can elevate the QBER rates for the QKD system. For instance, vibrations and noises from turbine operations and machinery can induce phase fluctuations in the quantum states used in QKD, leading to increased QBER. Similarly, noises from waterfall/spill and flow turbulence could impact the alignment and synchronization of the QKD system. Given the finite-size effect in the context of secure encryption, variations in QBER invariably gravitate towards the maximum bound of error. Hence, fluctuations introduced by the dam environment can be quite influential. The channel error model used for QKD simulations is described by Equation (4) [55]:

(4) $$e_{k}=p_{dc}+e_{mis}\left(1-e^{-\eta_{ch}k}\right)+\frac{p_{ap}D_{k}}{2},$$

where $e_{k}$ is the error rate for a coherent pulse with intensity *k*, $p_{dc}$ is the background noise rate of the detector (dark count rate), $p_{ap}$ is the after-pulse probability, and $D_{k}$ is the detection rate. $\eta_{ch}$ represents the loss due to the fiber optic cables and is given by $\eta_{ch}=10^{-0.2L/10}$, with *L* being the fiber length in km. The term $e_{mis}$ stands for the probability of error due to polarization changes in the channel and can be influenced by environmental factors.

**Figure 7:**
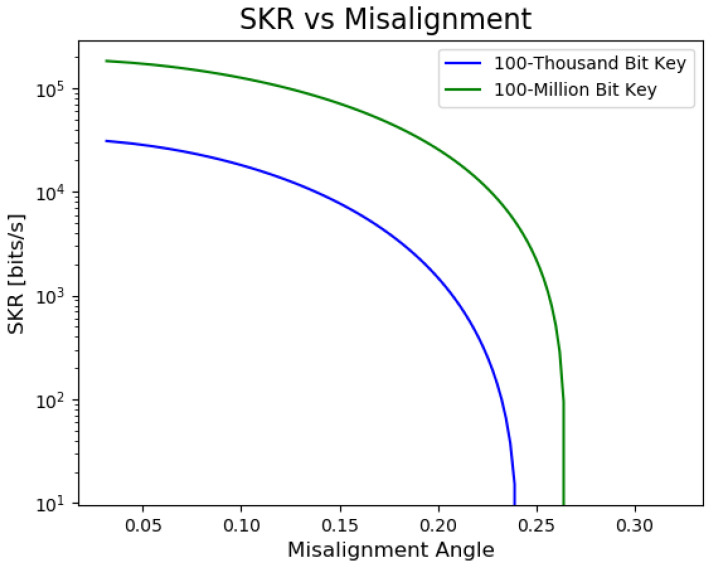
The secure key rate, depicted as a function of the misalignment angle, relies on Equations (2) and (4). Notably, commencing with a longer sifted key provides a buffer against higher degrees of polarization perturbation in the quantum channel.We note that we do not account for polarization drift in the simulation, but note that in practice, it is commonly actively stabilized in QKD experiments.

Given that turbine operations, with their frequencies typically around 1 Hz and 30 Hz (Table 4), can introduce vibrations of typically less than 1 mm in amplitude and that generators, operating at either 50 Hz or 60 Hz, induce similar amplitudes of vibrations, the environment’s vibrational noise becomes crucial. Environmental factors, from seismic activities to localized events like machinery operations, can further introduce vibrational noise that influences the polarization states in fiber optics, which are sensitive to such changes [58]. Understanding and mitigating these effects is pivotal for QKD. For instance, correlating the vibrational frequency and amplitude data with phase changes in the QKD system could enable real-time counteraction of potential misalignment errors. As observed in Figure 7, by choosing longer final key lengths, we can generate secure bits at a higher misalignment, an important consideration in a noise-prone dam environment. To ensure high secure key rates, polarization-based hydro QKD systems should adopt polarization stabilization techniques [59,60]. Typically, stabilization is achieved through feedback loops that monitor changes in the final state, enabling the sender to effectuate corrections. Given that numerous dams employ fiber-based sensors [61,62], integrating such vibrational and noise data into the stabilization algorithm offers a promising avenue to maintain optical alignment, optimizing the QKD system’s performance.

It is also worth noting here that recent advancements in QKD technologies, such as the integration of advantage distillation technology with decoy state QKD systems, have shown significant improvements in both the maximal transmission distance and the maximal tolerable error rate. This enhancement is particularly crucial for the extensive and remote operations characteristic of hydropower facilities, ensuring robust and secure communications over longer distances with higher reliability (see Li et al. [63]).

With measured SKR metrics in hand, the most appropriate cybersecurity strategy for QKD-secured hydro communications should be evaluated. This can be guided by the following principle. If the classical communication bandwidth needs for hydro are less than the SKR, then OTP may be employed (i.e., number of final key bits > number of classical bits requiring encryption). However, if the classical communication bandwidth needs exceed the link-specific SKR, then an alternative method must be employed, for example, where the same QKD key can be used to authenticate multiple messages, which can be accomplished as long as a QRNG supplies a new nonce [12]. Finally, regardless of the cryptography option above (OTP or authentication), the interface required to supply QKD keys to the user/application must be developed. This is dependent on the type, vendor, model of the user/application, and methods by which the device allows ingestion of external (i.e., QKD) key material. This final experiment will demonstrate the encryption/decryption of realistic hydropower command/control communications using QKD-supplied keys. Performance challenges include SKR changes with variations in the environment in which the subsystems of QKD are to operate. Dealing with various sources of noise (including those in Table 4) is of great importance in the successful generation of keys. For example, when both quantum and classical light are considered over the channel, a concern arises from “Raman noise”, which is unwanted light generated in the fiber material due to the scattering of stronger classical light. This effect is particularly pronounced when the wavelengths of the quantum and classical signals are closely multiplexed in wavelength. However, when they are in far-detuned bands, such as the quantum signal in the O band and the classical signal in the C band, the impact of Raman scattering is substantially mitigated [64,65]. Appropriate hardware choices can be made to better address the challenges and noise sources associated with the specific setting of the hydro facility. Although several QKD protocols exist, the well-established Bennet–Brassard protocol (BB84 protocol) makes for a suitable trial. Using the software, the dam communications can be interfaced with QKD keys. Similar experiments have been effectively performed to analyze and address implementation challenges facing the deployment of QKD systems in critical infrastructure, for example, as demonstrated in the recent field test of three QKD systems on a real-world electric utility optical fiber network [13], where one endpoint was a hydro/dam.

## 5. Conclusions and Outlook

Witnessing the overall growth trends of quantum technologies in solving energy infrastructure problems, the presented material introduced the specific use case of the hydroenergy sector. The preliminary discussions presented may help the creation of a more specialized “Quantum for Hydro” road map. Parameters that characterize the hydro/dam environment, as summarized in Table 4, are different from those found in a laboratory setting. Some of the parameters likely also differ from those encountered in the electric power grid substations where QKD has been demonstrated. Typical ambient real-world environmental conditions of importance to the performance of any technical measuring device include temperature, humidity, and various noise levels (electromagnetic, acoustic, wind, corrosion, contamination, etc.). QKD is built from sensitive optical and electronic components and devices, each with a set of specifications. Therefore, if, for example, these parameters are out of range, it could impact the rate of key generation. Consideration for the application of quantum sensing for environmental monitoring may also prove useful in conjunction with QKD. In compiling such a road map, important issues such as interoperability between QKD systems that operate with dissimilar implementations must be considered. In doing so, QKD standards by the ETSI Quantum-Safe Cryptography Working Group, and QKD network and QKD systems activities within ITU-T SG13 and SG17, respectively, will be put in perspective [13]. In closing our discussion, we anticipate that more research effort is needed to develop a comprehensive security ecosystem. Such efforts could, for example, include device-specific theoretical calculations for better adaptability and optimal performance, e.g., similar to those pertaining to UAV-based communication [66,67]. 

## Figures and Tables

**Table 1:** Brief list of basic cybersecurity issues and the role of QKD for dams.

Cybersecurity Issue	Description	Impact	Role of QKD
Remote Control System Attacks	Compromise of SCADA [6] systems controlling dam operations.	Dam failure, potential loss of life, and environmental damage.	Secure keys via QKD encrypt communication, thwarting unauthorized access to the control system.
Sensor Spoofing [7]	Interference with sensors, leading to inaccurate readings and unsafe operations.	Dam failure, potential loss of life, and environmental damage.	Secure or authenticated communication between sensors and control system, reveals data tampering.
Communication Interception [8]	Interception or injection of malicious commands in communication channels.	Dam failure, potential loss of life, and environmental damage.	Secure all communications, hindering interception or data tampering.
Denial of Service (DoS) Attacks [9]	Overloading communication channels or control systems.	Operational disruption leading to flooding or other issues.	Indirectly aids by protecting from vulnerabilities exploited in DoS.
Physical Security Breaches	Tampering of equipment or insertion of malicious hardware/software.	Dam failure, potential loss of life, and environmental damage.	Indirectly aids by strengthening overall cybersecurity infrastructure.
Supply Chain Attacks	Pre-installation compromise of hardware or software.	Compromised dam components leading to security breaches.	Indirectly aids by protecting from vulnerabilities exploited due to compromised components.
Insider Threats	Misuse of sensitive systems by authorized individuals.	Operational disruption or sabotage.	Secure communication between control systems and authorized personnel, preventing unauthorized access.

**Table 2:** QKD protocols and modalities (see, e.g., Diamanti et al. [21]) against hydropower system’s requirements.

QKD Modality	Hydropower System’s Requirements
BB84 Protocol (see, e.g., Nadal et al. [22] for other protocols)	Direct point-to-point setups for small-scale plants. Economically efficient for short (metro area) distances.
Decoy State QKD	Robust against photon number splitting attacks. Beneficial for medium to large-scale plants with potential eavesdropping threats. Usually combined with BB84 when photons are encoded in weak coherent pulses
Continuous-Variable QKD	Can be integrated with conventional signals on fiber networks. Requires direct trusted relay and quantum repeaters for long distances. Suitable for high transmission rate requirements.
MDI-QKD	Ideal for infrastructures at risk from sophisticated adversaries. Eliminates detector side-channel vulnerabilities at center detection node. May require higher initial investment for equipment.
Satellite-based QKD	Best for remote facilities over vast areas. Capital-intensive but offers broad coverage. Enables global-scale secure communications but at low rates. Requires clear sky conditions for optimal operations.
Other Considerations	Maintenance and operational costs. Scalability to future expansions. Interoperability with existing communication systems. Training and expertise requirements. Key management/revocation/lawful intercept requirements

**Table 3:** Basic noise sources in QKD systems in laboratory settings (see also [23,24,25,26,27]).

Noise Source	Description
Quantum Bit Error Ratio (QBER)	Represents the ratio of bits that are received in error. Though not a direct noise source, QBER quantifies the impact of various technical factors and imperfections in QKD systems.
Dark Counts	False counts arising in photon detectors due to thermal fluctuations or other non-signal measurement events.
Dead Time	Time taken by a detector to recover after detecting a photon. Photons arriving during this interval can lead to loss.
Detector Jitter	Uncertainty in a detector’s time response when it receives a signal, arising from electronic and photonic fluctuations.
Beam Splitting/Coupling Inefficiencies	Imperfections in beam splitters or inefficient coupling into optical fibers leading to photon loss.
Fiber or Channel Attenuation	Losses in the optical channel or the transmission fiber.
Multi-Photon Emissions	Occurrences when sources produce multi-photon pulses, introducing vulnerabilities and noise.
Phase Fluctuations	In protocols like Differential Phase Shift QKD, phase fluctuations in transmission fiber can cause errors.
Timing Jitter/Synchronization	Uncertainty or variations in the timing of a system’s clock or reference signal, affecting synchronization.
Quantum State Preparation	Imperfections in preparing quantum states for example, specific polarization state encoding.
Spatial Mode Mismatches	Mismatches when transmitting quantum states over channels, leading to decreased detection probabilities.
Back Reflections/Scattering	Reflections from interfaces or scattering within components introducing noise photons.

**Table 4:** Typical noise sources in a dam environment and corresponding sensors with quantitative descriptions (refer to Ouellet et al. [28] for monitoring relevant noise sources, and other works [29,30,31,32] for specific examples of noise frequency and amplitude).

Noise Source	Description	Typical Sensor
Turbine Operations	Noise from turbine movement both in air and underwater. Frequencies f∼(0.5–30) Hz with amplitudes ≲1 mm.	Hydrophone (underwater), Microphone (airborne)
Gates and Valves	Noise due to dam gate or spillway operations. Varies based on size and operation speed.	Vibration sensors, Microphone
Pumps and Machinery	Noise from operational machinery. Typically f∼(10–200) Hz.	Microphone, Vibration sensors
Flow Turbulence	Noise from rapid and turbulent water flow. f∼(1–100) Hz.	Hydrophone
Waterfall/Spill	Noise due to water spillage. Frequency depends on water volume and height of fall.	Hydrophone, Microphone
Bubble Formation	Noise due to bubble formation and collapse. f∼(5–50) Hz.	Hydrophone
Transformer Operations	Buzzing or humming from transformers. Typically at 50 Hz or 60 Hz.	Magnetic field sensors, Microphone
High Voltage Equipment	Noise from insulator discharges. Broadband noise typically spanning 10 Hz to 1 kHz.	Electromagnetic sensors, Microphone
Vibration	Vibrations inherent to dam structures. Spanning from very low frequencies (<1 Hz) due to seismic activities to high frequencies (>100 Hz) from machinery operations.	Accelerometers, Vibration sensors
Thermal Expansion/Contraction	Noise from temperature-induced structural changes. Frequency varies based on structure size and material.	Vibration sensors, Microphone
Wildlife Activities	Sounds from local fauna. Frequencies are species-specific, ranging broadly from 1 Hz to 10 kHz.	Microphone, Hydrophone
Weather Patterns	Noise from atmospheric disturbances, thunder, tornado. Broad frequency range from <1 Hz (thunder rumble) to >10 kHz (lightning crack).	Wind sensors, Microphone
Vehicle Traffic	Noise from vehicular activities. Frequencies range from 20 Hz (engine hum) to 2 kHz (horn).	Accelerometers, Vibration sensors, Microphone
Construction/Maintenance	Noise from maintenance or construction work. Broad frequency range depending on tools and machinery.	Accelerometers, Microphone, Vibration sensors
Temperature Fluctuations	Ambient temperature changes affecting equipment. Changes can cause material contractions or expansions leading to noise.	Thermocouples, Infrared sensors
Moisture/Condensation	Moisture interference with equipment. Can cause electrical noises or material deformations.	Humidity sensors, Moisture meters

**Table 5:** Basic post-processing steps in QKD (see Figure 5, and R. Wolf [49] for further reading).

Step	Description/Equation
Error Estimation	$\mathrm{QBER}=\frac{\mathrm{number}\;\mathrm{of}\;\mathrm{error}\;\mathrm{bits}}{\mathrm{total}\;\mathrm{number}\;\mathrm{of}\;\mathrm{bits}\;\mathrm{exchanged}}$
Information Reconciliation	Uses error-correcting codes to rectify key discrepancies. The Cascade protocol is popular; it entails key division, shuffling, and parity comparison.
Privacy Amplification	Aims to eliminate any eavesdropper’s partial information. Typically employs universal hash functions, represented as: $k=f(k_{\mathrm{raw}})$.
Key Sifting	Particularly relevant in the BB84 protocol. Alice and Bob publicly disclose the bases chosen for each qubit. Qubits with differing bases are discarded.
Authentication	Confirms genuine communication between Alice and Bob. Utilizes classical authentication methods in tandem with previously shared secret keys.

## Data Availability

Not applicable.

## Appendix A. Simplified Examples of Classical and Quantum Keys for Encryption

Given the cross-disciplinary nature of this discussion, we provide a brief technical introduction to quantum cryptography. Below, starting with a simple classical encryption using the (Vernam cipher) one-time-pad (OTP) and BB84 protocol key generation, we discuss how a simple message may be encrypted using QKD keys via post-processing protocol. Other simplified QKD experiments and related theories include the work by Bloom et al. [68].

### Appendix A.1. Classical Key Encryption

As an example of classical encryption, suppose we would like to securely communicate the message “dam” between an infrastructure point and a remote control unit. Let us write it in binary representation based on ASCII values. In the ASCII standard, each character is represented using a unique seven-bit binary code. For instance, the binary representation for the character “d” is obtained by translating its ASCII representation (100) to a seven-bit binary string, yielding 1100100. Therefore,

(A1) dam→110010011000011101101.

The one-time-pad (OTP) is a symmetric key encryption technique known to be unbreakable when used correctly. It applies a key (usually a random sequence of bits) to the message via a bitwise operation, typically XOR, to produce the ciphertext. To assure security using the OTP, the key should be as long as the plaintext, used only once, and kept secret. As our example key, let us take the word “key”. The binary representation for “key” is:

(A2) key→110101111001011111001.

Let us now perform the XOR operation, which is defined as follows. Given two binary values *A* and *B*, the XOR (exclusive or) operation returns a value of 1 if the bits being compared are different, and 0 if they are the same. Formally:

(A3) A⊕B=C,

and, if we XOR the result *C* with *B*, we retrieve the original value *A*:

(A4) C⊕B=A.

Thus, for our “(dam⊕key)→ciphertext” operation, we write:

(A5) $$\begin{array}{cc} 1100100\;\left(d\right)\;\;\; & ⊕\;\;\;1101011\;\left(k\right)\to 0001111, \end{array}$$

(A6) $$\begin{array}{cc} 1100001\;\left(a\right)\;\;\; & ⊕\;\;\;1100101\;\left(e\right)\to 0000100, \end{array}$$

(A7) $$\begin{array}{cc} 1101101\;\left(m\right)\;\;\; & ⊕\;\;\;1111001\;\left(y\right)\to 0010100. \end{array}$$

Thus, the encrypted message is:

(A8) cyphertext:000111100001000010100.

To decipher the ciphertext produced using the OTP, we reapply the same XOR operation with the same key. This returns us to the original plaintext. Using our previously derived ciphertext and the key:

(A9) $$\begin{array}{cc} 0001111\;\;\; & ⊕\;\;\;1101011\;\left(k\right)\to 1100100\;\left(d\right), \end{array}$$

(A10) $$\begin{array}{cc} 0000100\;\;\; & ⊕\;\;\;1100101\;\left(e\right)\to 1100001\;\left(a\right), \end{array}$$

(A11) $$\begin{array}{cc} 0010100\;\;\; & ⊕\;\;\;1111001\;\left(y\right)\to 1101101\;\left(m\right). \end{array}$$

Thus, when deciphered, the ciphertext using the key gives us back the original message:

(A12) plaintext:110010011000011101101→“dam”.

This illustrates the reversible nature of the XOR operation; the encryption and decryption processes are effectively the same operation. The generation of classical keys, especially for cryptographic purposes, is more intricate than just using a straightforward binary representation of a word, as we simplistically did above. The strength of cryptographic systems often hinges on the quality of the keys and the randomness or unpredictability of key generation. Classical keys may be generated via true, as well as pseudorandom random number generators, though the latter does not provide much security. Key management practices, including generation, storage, distribution, rotation, and disposal, are vital.

### Appendix A.2. Quantum Key Encryption

The above encryption can also be performed using the key generated via QKD. The communicating parties (Alice and Bob) could use a QKD protocol such as BB84 to generate a shared, secret random bit-string. This process involves sending quantum states (e.g., photon polarizations) between Alice and Bob and performing post-processing following the steps in Figure 5 and Table 5 (see R. Wolf [49] for further reading). Alice can now use the shared key from the QKD process as the OTP key to XOR with her message, “dam”, in binary.

dam→110010011000011101101

Suppose the QKD-generated key is $K=k_{1}\;k_{2}\;k_{3}$ (where each $k_{i}$ is seven bits in our toy example). She then XORs her message with this key to obtain the ciphertext:

$$\begin{array}{cc} 1100100⊕k_{1} & =c_{1}\\ 1100001⊕k_{2} & =c_{2}\\ 1101101⊕k_{3} & =c_{3} \end{array}$$

The encrypted message is then $c_{1}\;c_{2}\;c_{3}$. For decryption, Bob uses the same QKD-generated key to XOR with the received ciphertext to retrieve the original message.

**Step 1: Preparation and Transmission by Alice**
  - Alice randomly selects bits and their corresponding bases. The bases can be:
    – Rectilinear, represented as Z-basis: |0〉 and |1〉.
    – Diagonal, represented as X-basis: $|+\rangle =\frac{|0\rangle +|1\rangle }{\sqrt{2}}$ and $|-\rangle =\frac{|0\rangle -|1\rangle }{\sqrt{2}}$.
  - For demonstration, consider the binary of “dam”: 1100100 1100001 1101101. Alice selects the first three bits: 110.
    – For the first bit (1), Alice chooses the rectilinear basis and sends |1〉.
    – For the second bit (1), Alice chooses the diagonal basis and sends |−〉.
    – For the third bit (0), Alice chooses the rectilinear basis and sends |0〉.
**Step 2: Measurement by Bob**
  - Bob randomly selects a basis for each received qubit and performs a measurement.
    – For the first qubit, he chooses rectilinear and measures 1.
    – For the second qubit, he chooses rectilinear (a mismatch with Alice) and obtains a random result, say 0.
    – For the third qubit, he chooses rectilinear and measures 0.
**Step 3: Basis Discussion**
  - Alice and Bob publicly disclose the bases they used.
  - They compare their choices:
    – For the first bit, both chose rectilinear—they retain Bob’s result.
    – For the second bit, they used different bases—they discard Bob’s result.
    – For the third bit, both chose rectilinear—they retain Bob’s result.
  - Their resulting raw key is now 10.
**Step 4: Error Estimation**
  - A subset of the raw key is selected for error testing.
  - They compare their respective bits in this subset publicly.
  - Calculate QBER = (number of errors in subset)/(size of subset).
  - If QBER exceeds a threshold, the protocol is aborted due to potential eavesdropping.
**Step 5: Privacy Amplification**
  - Aims to reduce any potential eavesdropper’s information to an insignificant level.
  - Two-universal hash functions might be applied to the key to produce a shorter, more secure key. For example, take the raw key to be 1011010101. Consider a very basic hash function defined as follows:
    – Break the string into groups of 2.
    – For each group:
      ∗ If it is 00, it maps to 0.
      ∗ If it is 01 or 10, it maps to 1.
      ∗ If it is 11, it maps to 0.
    – Given the original key, 1011010101, applying the hash function produces 10111, which is shorter than the original key as a result of a specific transformation.
  - Classical error-correcting codes can be used to rectify errors introduced by the quantum channel. For example, consider the Hamming(7,4) code [69]:
    – Designed to encode four bits of data into seven bits by adding three parity bits.
    – Given a four-bit data ‘1101’, encoding adds parity bits to produce 0 0 1 0 1 1 0.
    – If an error flips the sixth bit during transmission, we receive 0 0 1 0 1 0 0.
    – The error is detected and corrected using the parity bits, restoring the original encoded string.

Our plaintext message “dam” has a length of 21 bits, as noted above. Due to the probabilistic nature of quantum measurements and the random choice of bases, typically only around 50% of the initially sent photons contribute to the raw key post key sifting for the BB84 protocol. So, if Alice wants to ensure a shared secret key of length 21 bits (to match the plaintext message length), she will need to initiate the process with more than 42 encoded photons. Thus, the process requires the transmission of a greater number of photons than the intended message length due to the key sifting process and potential eavesdropping checks.

## Appendix B. Glossary

**Table A1:** Glossary of Terms and Definitions.

**Term**	**Definition**
*a*	Fiber’s Attenuation: Represented in units of dB/km, it is the property of the optical fiber that quantifies the loss of signal strength per unit length of fiber.
Alice and Bob	Conventionally used names to denote the sender and receiver in cryptographic communications, including in QKD systems.
Attenuation	The reduction of signal strength as it travels through a medium, such as an optical fiber, due to absorption, scattering, and other loss mechanisms.
BB84	A quantum key distribution protocol developed in 1984 by Bennett and Brassard, using two non-orthogonal bases [56].
Binary Entropy Function	A function quantifying the maximum possible information about the total key based on shared bits between parties. Typically denoted as H(p), it represents the uncertainty of a binary random variable and is defined as [49,56]: $H\left(p\right)=-p\log_{2}\left(p\right)-\left(1-p\right)\log_{2}\left(1-p\right),$ where *p* is the probability of one of the two outcomes (e.g., a bit being 1). Consequently, 1−p is the probability of the other outcome (the bit being 0). *H* is defined for 0≤p≤1 with a maximum of 1, which occurs when p=0.5, indicating maximum uncertainty (i.e., both outcomes are equally probable). When p=0 or p=1, H(p)=0, there is no uncertainty. In our work, Equation (3) yields the error rate in a specific basis (*x* basis) and represents the maximum possible information rate that can be deduced about the total key based on shared bits for error estimation.
Channel Loss Parameter ($\eta_{ch}$)	A dimensionless parameter derived from the total optical loss (γ) and representing the linear loss.
Coherent States	A coherent quantum state |α〉 is defined as: $|\alpha \rangle =e^{-\frac{{\left|\alpha \right|}^{2}}{2}}\sum_{n=0}^{\infty }\frac{\alpha^{n}}{\sqrt{n!}}|n\rangle ,$ where α is a complex number and |n〉 are the photon number (Fock) states [70]. Such states play a fundamental role in quantum optics due to their semi-classical nature and are crucial in various quantum communication protocols, including QKD [49].
Continuous Variable-Quantum Key Distribution (CV-QKD)	CV-QKD employs continuous quantum variables such as the quadratures of the electromagnetic field to encode information. The most common CV-QKD protocols are based on coherent states using Gaussian modulation of amplitude and phase, and they use homodyne or heterodyne detection for the decoding process. This approach has the advantage of being compatible with conventional telecom technology, potentially allowing for more straightforward integration into existing networks. However, it typically makes more assumptions that detector noise can be “trusted”, and calibrated away.
Dark Counts	False positive detector counts, usually due to detector noise.
Decoy State QKD	A variation of the BB84 QKD protocol which employs additional signal states to improve security against photon number splitting attacks.
Discrete Variable (DV) QKD	Discrete Variable (DV) QKD leverages the quantum properties of individual photons to distribute shared symmetric random numbers. It typically operates using polarization or phase encoding schemes to encode the random quantum information. Protocols such as BB84, B92, and SARG04 are well-known in the DV-QKD [22,52].
Error Correction	A process to identify and correct errors in the quantum key transmission.
Error Rate in the X Basis ($\phi_{x}$)	A parameter representing the rate of error in the x basis of the key during QKD operations.
Error Reconciliation	A procedure in QKD to correct any discrepancies in the key between the two parties.
Eve	Conventionally used name to denote a potential attacker trying to gain unauthorized access to the quantum transmissions. All errors are typically attributed to her.
Fiber-Based Loss	Refers to the loss of signal in optical fibers, affecting the transmission of quantum signals.
Fiber Length (*L*)	The physical distance covered by the optical fiber, usually represented in kilometers.
Finite Size Effects	Describes the effects or limitations of having a finite number of measured signals in QKD.
Homodyne Detection	A technique used in quantum cryptography for measuring a quantum signal through interference with a local oscillator using balanced difference detection.
Hydropower	The generation of power through the use of the gravitational force of falling or fast-running water.
Information Theoretical Security (ITS)	A security paradigm that assures confidentiality regardless of the computational resources of an adversary.
Leakage During Error Correction (${\mathrm{Leak}}_{EC}$)	The segment of key information that might be exposed to any potential adversary during error correction procedures in the QKD protocol.
Misalignment Angle	In polarization encoded QKD, refers to the variation in the angle of the initial polarization state, which can be caused by factors such as thermal fluctuations or physical stress on the fiber.
One-Time Pad (OTP)	A method of encryption where a message is combined with a one-time-use key using exclusive OR (XOR) operation.
Phase Modulation	The modulation of the phase of a carrier signal to encode information, often used in QKD systems to encode quantum information.
Avalanche Photo Detectors (APD)	Photodetectors that can detect low-intensity light down to single photons, often used in QKD receivers.
Polarization	Refers to the orientation of oscillations in electromagnetic waves, used to encode information in quantum states in the context of QKD.
Quantum Bit Error Ratio (QBER)	The ratio of errors that occur during quantum transmission.
Quantum Cryptography (QC)	A method of cryptography with a trust anchor rooted in quantum mechanics principles.
Quantum Key Distribution (QKD)	A cryptographic protocol based on quantum mechanics to securely distribute random private keys between two parties.
Secure Key Length (*L*)	The length of the secure key.
Secure Key Rate (SKR)	The rate at which a QKD system can produce secure shared private keys, influenced by factors like distance and error rate. A metric to evaluate the performance of a QKD system
Secure Rate Formula	A mathematical representation of the rate at which a QKD system can generate secure keys.
Total Optical Loss (γ)	Represented in units of dB, it measures the total loss in the system, arising due to the fiber’s attenuation (*a*), imperfect components, and the length of the fiber (*L*).

## References

[1] Rass S., Schauer S., König S., Zhu Q. (2020). Cyber-Security in Critical Infrastructures.

[2] Whyatt M., Whyatt M.V., Thorsen D.E., Watson M.D., Ham K.D., Pederson P.A., McKinnon A.D., DeSomber K.R. (2021). Toward a Resilient Cybersecure Hydropower Fleet: Cybersecurity Landscape and Roadmap 2021.

[3] (2019). Dams Sector Landscape.

[4] Singh P., Singh S., Vardhan S., Patnaik A. (2020). Sustainability of maintenance management practices in hydropower plant: A conceptual framework. Mater. Today Proc..

[5] Ratnam E.L., Baldwin K.G., Mancarella P., Howden M., Seebeck L. (2020). Electricity system resilience in a world of increased climate change and cybersecurity risk. Electr. J..

[6] Alrefaei A.S. An Overview of Securing SCADA Systems: The Gap in the Physical Security Measure. Proceedings of the 2022 Fifth National Conference of Saudi Computers Colleges (NCCC).

[7] Urbina D.I., Giraldo J.A., Cardenas A.A., Tippenhauer N.O. (2016). Survey and new directions for physics-based attack detection in process control systems. Proceedings of the IFIP Annual Conference on Data and Applications Security and Privacy.

[8] Chen T.M., Abu-Nimeh S. (2011). Lessons from Stuxnet. Computer.

[9] Lee R.M., Assante M.J., Conway T. (2016). Analysis of the Cyber Attack on the Ukrainian Power Grid.

[10] Passian A., Imam N. (2019). Nanosystems, edge computing, and the next generation computing systems. Sensors.

[11] Farahi R., Passian A., Tetard L., Thundat T. (2012). Critical issues in sensor science to aid food and water safety. ACS Nano.

[12] Alshowkan M., Evans P.G., Starke M., Earl D., Peters N.A. (2022). Authentication of smart grid communications using quantum key distribution. Sci. Rep..

[13] Evans P.G., Alshowkan M., Earl D., Mulkey D., Newell R.T., Peterson G., Safi C.L., Tripp J.L., Peters N.A. (2021). Trusted Node QKD at an Electrical Utility. IEEE Access.

[14] Grice W., Evans P., Pooser R. (2013). Quantum Key Distribution for the Smart Grid. IEEE Vision for Smart Grid Communications: 2030 and Beyond.

[15] Kuruganti T. (2014). Quantum Key Distribution Applicability to Smart Grid Cybersecurity Systems. Internal Technical Report, ORNL. https://www.ornl.gov/research-library#stq=%22Quantum%20Key%20Distribution%20Applicability%20to%20Smart%20Grid%20Cybersecurity%20Systems.%22&stp=1.

[16] Dunjko V., Wallden P., Andersson E. (2014). Quantum Digital Signatures without Quantum Memory. Phys. Rev. Lett..

[17] Chen Y.A., Zhang A.N., Zhao Z., Zhou X.Q., Lu C.Y., Peng C.Z., Yang T., Pan J.W. (2005). Experimental Quantum Secret Sharing and Third-Man Quantum Cryptography. Phys. Rev. Lett..

[18] Long G.l., Deng F.g., Wang C., Li X.h., Wen K., Wang W.y. (2007). Quantum secure direct communication and deterministic secure quantum communication. Front. Phys. China.

[19] Cao Z., Lu Y., Chai G., Yu H., Liang K., Wang L. (2023). Realization of Quantum Secure Direct Communication with Continuous Variable. Research.

[20] Rothe S., Besser K.L., Krause D., Kuschmierz R., Koukourakis N., Jorswieck E., Czarske J.W. (2023). Securing Data in Multimode Fibers by Exploiting Mode-Dependent Light Propagation Effects. Research.

[21] Diamanti E., Lo H.K., Qi B., Yuan Z. (2016). Practical challenges in quantum key distribution. NPJ Quantum Inf..

[22] Nandal R., Nandal A., Joshi K., Rathee A.K. (2021). A survey and comparison of some of the most prominent QKD protocols. SSRN Electron. J..

[23] Scarani V., Bechmann-Pasquinucci H., Cerf N.J., Dušek M., Lütkenhaus N., Peev M. (2009). The security of practical quantum key distribution. Rev. Mod. Phys..

[24] Gobby C., Yuan Z., Shields A. (2004). Quantum key distribution over 122 km of standard telecom fiber. Appl. Phys. Lett..

[25] Yuan Z., Kardynal B., Sharpe A., Shields A. (2007). High speed single photon detection in the near infrared. Appl. Phys. Lett..

[26] Rosenberg D., Peterson C.G., Harrington J.W., Rice P.R., Dallmann N., Tyagi K.T., McCabe K.P., Nam S., Baek B., Hadfield R.H. (2005). Practical long-distance quantum key distribution system using decoy levels. New J. Phys..

[27] Hiskett P.A., Rosenberg D., Peterson C.G., Hughes R.J., Nam S., Lita A.E., Miller A.J., Nordholt J.E. (2007). Long-distance quantum key distribution in optical fibre. New J. Phys..

[28] Ouellet S.M., Dettmer J., Olivier G., DeWit T., Lato M. (2022). Advanced monitoring of tailings dam performance using seismic noise and stress models. Commun. Earth Environ..

[29] Antonovskaya G., Kapustian N., Basakina I., Afonin N., Moshkunov K. (2019). Hydropower Dam State and Its Foundation Soil Survey Using Industrial Seismic Oscillations. Geosciences.

[30] Baron P., Kočiško M., Hlavatá S., Franas E. (2022). Vibrodiagnostics as a predictive maintenance tool in the operation of turbo generators of a small hydropower plant. Adv. Mech. Eng..

[31] Mohanta R.K., Chelliah T.R., Allamsetty S., Akula A., Ghosh R. (2017). Sources of vibration and their treatment in hydro power stations-A review. Eng. Sci. Technol. Int. J..

[32] Quaranta E., Müller G. (2021). Noise Generation and Acoustic Impact of Free Surface Hydropower Machines: Focus on Water Wheels and Emerging Challenges. Int. J. Environ. Res. Public Health.

[33] Philippe S., d’Errico F. (2020). A physical unclonable neutron sensor for nuclear arms control inspections. Sci. Rep..

[34] Ijaz S., Rana A.S., Ahmad Z., Zubair M., Massoud Y., Mehmood M.Q. (2022). The Dawn of Metadevices: From Contemporary Designs to Exotic Applications. Adv. Devices Instrum..

[35] Kwek L.C., Cao L., Luo W., Wang Y., Sun S., Wang X., Liu A.Q. (2021). Chip-based quantum key distribution. AAPPS Bull..

[36] Zhang Z., Xu N., Huang Z., Lai J., Liu J., Deng G., Wang X., Zhao W. (2023). High-Sensitivity Force Sensors Based on Novel Materials. Adv. Devices Instrum..

[37] Wang M., Zhang F. (2023). Squeezing for cosmic symphony. AAPPS Bull..

[38] Lawrence J., Hollern J., Geddes B., Geddes B., Freeman S., Reif M., Reiger C. (2020). Fossil Power Plant Cyber Security Life-Cycle Risk Reduction, a Practical Framework for Implementation.

[39] Bharani P., Chandra K., Potnuru D. (2021). A nonlinear load frequency controller for hydropower plants. Int. J. Ambient. Energy.

[40] Wang S., Yin Z.Q., He D.Y., Chen W., Wang R.Q., Ye P., Han Z.F. (2022). Twin-field quantum key distribution over 830-km fibre. Nat. Photonics.

[41] Liao S., Cai W.Q., Liu W.Y., Zhang L., Li Y., Ren J.G., Pan J.W. (2017). Satellite-to-ground quantum key distribution. Nature.

[42] Yin J., Li Y.H., Liao S.K., Yang M., Cao Y., Zhang L., Pan J.W. (2020). Entanglement-based secure quantum cryptography over 1120 kilometres. Nature.

[43] Sharma P., Agrawal A., Bhatia V., Prakash S., Mishra A.K. (2021). Quantum Key Distribution Secured Optical Networks: A Survey. IEEE Open J. Commun. Soc..

[44] Qi B., Zhu W., Qian L., Lo H.K. (2010). Feasibility of quantum key distribution through a dense wavelength division multiplexing network. New J. Phys..

[45] Scherer A., Sanders B.C., Tittel W. (2011). Long-distance practical quantum key distribution by entanglement swapping. Opt. Express.

[46] Lo H.K., Ma X., Chen K. (2005). Decoy state quantum key distribution. Phys. Rev. Lett..

[47] Lütkenhaus N. (2000). Security against eavesdropping in quantum cryptography. Phys. Rev. A.

[48] Makarov V., Khan J. (2006). Optical attacks on practical continuous-variable quantum key distribution systems (or ‘how to hack a quantum cryptosystem’). Opt. Lett..

[49] Wolf R. (2021). Quantum Key Distribution.

[50] Pljonkin A., Singh P.K. The Review of the Commercial Quantum Key Distribution System. Proceedings of the 2018 Fifth International Conference on Parallel, Distributed and Grid Computing (PDGC).

[51] Moreno Escobar J.J., Morales Matamoros O., Tejeida Padilla R., Lina Reyes I., Quintana Espinosa H. (2021). A comprehensive review on smart grids: Challenges and opportunities. Sensors.

[52] Kong P.Y. (2020). A review of quantum key distribution protocols in the perspective of smart grid communication security. IEEE Syst. J..

[53] Gopstein A., Nguyen C., O’Fallon C., Hastings N., Wollman D. (2021). NIST Framework and Roadmap for Smart Grid Interoperability Standards. National Institute of Standards and Technology Special Publication (NIST SP), Release 2 (Latest 4). https://nvlpubs.nist.gov/nistpubs/SpecialPublications/NIST.SP.1108r4.pdf.

[54] US Department of Energy (2001). 21 Steps to Improve Cyber Security of SCADA Network.

[55] Lim C., Curty M., Walenta N., Xu F., Zbinden H. (2014). Concise security bounds for practical decoy-state quantum key distribution. Phys. Rev..

[56] Wilde M.M. (2017). Preface to the Second Edition. Quantum Information Theory.

[57] Moschandreou E., Rollick B.J., Qi B., Siopsis G. (2021). Experimental decoy-state Bennett-Brassard 1984 quantum key distribution through a turbulent channel. Phys. Rev. A.

[58] Ding Y.Y., Chen H., Wang S., He D.Y., Yin Z.Q., Chen W., Zhou Z., Guo G.C., Han Z.F. (2017). Polarization variations in installed fibers and their influence on quantum key distribution systems. Opt. Express.

[59] Mekhtiev E., Gerasin I., Rudavin N., Duplinsky A., Kurochkin Y. (2021). Polarization control algorithm for QKD systems. Proc. J. Phys. Conf. Ser..

[60] Wu G., Chen J., Li Y., Zeng H. (2006). Stable polarization-encoded quantum key distribution in fiber. arXiv.

[61] Inaudi D., Blin E.R. Monitoring Dams with Distributed Fiber Optic Sensing. Proceedings of the International Conference on Dam Engineering.

[62] de la Torre O., Floris I., Sales S., Escaler X. (2021). Fiber Bragg Grating Sensors for Underwater Vibration Measurement: Potential Hydropower Applications. Sensors.

[63] Li H.W., Zhang C.M., Jiang M.S., Cai Q.Y. (2022). Improving the performance of practical decoy-state quantum key distribution with advantage distillation technology. Commun. Phys..

[64] Peters N.A., Toliver P., Chapuran T.E., Runser R.J., McNown S.R., Peterson C.G., Rosenberg D., Dallmann N., Hughes R.J., McCabe K.P. (2009). Dense wavelength multiplexing of 1550 nm QKD with strong classical channels in reconfigurable networking environments. New J. Phys..

[65] Chapuran T.E., Toliver P., Peters N.A., Jackel J., Goodman M.S., Runser R.J., McNown S.R., Dallmann N., Hughes R.J., McCabe K.P. (2009). Optical networking for quantum key distribution and quantum communications. New J. Phys..

[66] Zhu Q., Zhao Z., Mao K., Chen X., Liu W., Wu Q. (2021). A Real-Time Hardware Emulator for 3D Non-Stationary U2V Channels. IEEE Trans. Circuits Syst. I Regul. Pap..

[67] Hua B., Ni H., Zhu Q., Wang C.X., Zhou T., Mao K., Bao J., Zhang X. (2023). Channel Modeling for UAV-to-Ground Communications With Posture Variation and Fuselage Scattering Effect. IEEE Trans. Commun..

[68] Bloom Y., Fields I., Maslennikov A., Rozenman G.G. (2022). Quantum Cryptography–A Simplified Undergraduate Experiment and Simulation. Physics.

[69] Siehler J.A. The Hamming(7,4) Code. *Wolfram Demonstrations Project*, 7 March 2011. http://demonstrations.wolfram.com/TheHamming74Code/.

[70] Loudon R. (2000). The Quantum Theory of Light.

